# Perspectives on the Role and Synergies of Architecture and Social and Built Environment in Enabling Active Healthy Aging

**DOI:** 10.1155/2016/6189349

**Published:** 2016-08-30

**Authors:** Evangelia Chrysikou, Richard Rabnett, Chariklia Tziraki

**Affiliations:** ^1^Faculty of the Built Environment, UCL Bartlett School of Architecture, 140 Hampstead Rd., London NW1 2BX, UK; ^2^AM-GAR, Hashdera Hamerkazit 15, Ligad 1, 7173003 Modi'in, Israel; ^3^MELABEV-Community Elders Club, Research and Evaluation Department and Hebrew University of Jerusalem, Israel Gerontological Data Center, 9190501 Mount Scopus, Israel

## Abstract

Research has demonstrated that enabling societal and physical infrastructure and personal accommodations enhance healthy and active aging throughout the lifespan. Yet, there is a paucity of research on how to bring together the various disciplines involved in a multidomain synergistic collaboration to create new living environments for aging. This paper aims to explore the key domains of skills and knowledge that need to be considered for a conceptual prototype of an enabling educational process and environments where healthcare professionals, architects, planners, and entrepreneurs may establish a shared theoretical and experiential knowledge base, vocabulary, and implementation strategies, for the creation of the next generation of living communities of active healthy adults, for persons with disabilities and chronic disease conditions. We focus on synergistic, paradigmatic, simple, and practical issues that can be easily upscaled through market mechanisms. This practical and physically concrete approach may also become linked with more elaborate neuroscientific and technologically sophisticated interventions. We examine the domains of knowledge to be included in establishing a learning model that focuses on the still-understudied impact of the benefits toward active and healthy aging, where architects, urban planners, clinicians, and healthcare facility managers are educated toward a synergistic approach at the operational level.

## 1. Introduction

According to the World Health Organization (WHO), physical and social environments are key determinants in maintaining an autonomous, meaningful life along the aging process [1]. Research from epidemiological studies and social sciences has provided sufficient evidence that maintaining health, independency, and autonomy would imbue longevity with wellbeing, a more meaningful life span, and lower healthcare costs. Urban planning, architecture of healthcare facilities that promote healing, and domestic buildings supporting autonomy are important elements in enabling a more active and healthy aging process throughout the life span. Yet, in practice, the fields of traditional healthcare and architecture or urban planning seldom cross paths in either the academic curricula, the research arena, or the actual execution of theory and evidence-based research and planning of these physical spaces within which individuals and societies age [2]. This paper aims to outline the domains of a new model of multidisciplinary learning which takes place in face-to-face interactions with all the interested parties: architects, healthcare providers and managers, and planners. Aging is not a disease, but rather a normal and valued part of the life course, as defined by the WHO [1, p. 103]. In this paper, we briefly present the theoretical complexity and the multidisciplinary nature involved in the planning of the healthcare facilities and domestic living spaces. The three elements comprising this planning: the healthcare framework, the social context and the physical milieu, and their interrelationships. We then present the argument that by examining how to intergrade these three elements in a potentially synergistic enabling educational process would lead the participants to ponder on the integration of these elements in real market scenarios. Our approach is informed by the synergies groups generated by the EIP AHA [3] and planned activities of our commitment to A2 group of the EIP AHA [4].

## 2. Theoretical Framework

### 2.1. Health and Society and Space: An Integrated Perspective

Epidemiological studies have identified a strong link between health status and societal domains [5]. The healthcare system and its practitioners are aiming to use this evidence-based knowledge in supporting and advocating policy at the local level for improvement of the social determinants of health including the built environment [6]. Hillier and Hanson's [7] work has contributed significantly to the interconnection and synergies of the built environment, society, and space, arguing that there is a social logic of space and that the linkage is cross-cultural and not age specific [8]. Thus, the link between health and society has been established through public health, and the link between society and space has been established through space syntax [9]. The theory of salutogenesis [10] joints all three domains bridging health and physical space. Salutogenesis, derived from medical sociology, was conceived by Antonovsky [11] who looked at the relationship between stress and wellbeing, through the “Sense of Coherence.” Antonovsky's salutogenesis theory focuses on the factors and mechanisms that promote health, not on illness [12]. The multidisciplinary and the intercultural application of the “Sense of Coherence,” an essential element of the salutogenic theory [13], has influenced a growing body of research based design framework of the built environment, creating a platform for a creative dialogue between healthcare and architecture [14–16]. Although Antonovsky did not aim directly at the spatial elements, which are considered part of the physical pillar [17] that lead to increase somebody's “Sense of Coherence” [18], his theories has prompted the generation of a variety of fields, including practitioners of medical architecture, salutogenic design or design and health field [19–21], and Healing Gardens [22].

**Figure 1:**
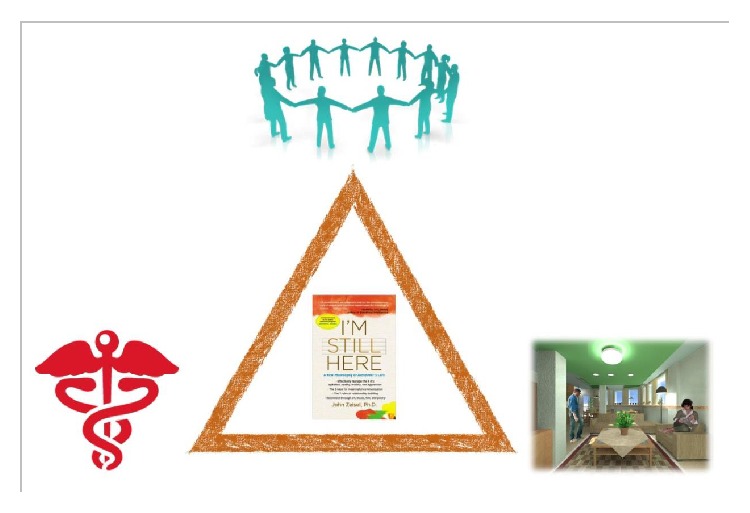
Visual interpretation of Zeisel's three key elements in the treatment and care of people with dementia—the medication, the human interaction, and the physical environment.

The work of Zeisel on Alzheimer's [23] is a paradigmatic example of the three pillars of Salutogenesis connecting health, space, and society and stresses the benefits deriving from their synergy, as depicted in Figure 1.

Zeisel advocates that although there are three key elements in the treatment and care of people with Alzheimer's, that is, the medication, the human interaction, and the physical environment, funding and research concentrate on the first, reducing considerably the resources allocated to the other two. This disproportionate allocation of resources happens despite the fact that space and human care may have significant, sustained, and immediate impact on health status of dementia person, when compared to pharmaceuticals where progress still needs to be made [23].

In this paper, we aim to highlight the need for synergy between healthcare systems, the built environment, as well as their social context including public and private domains and urban setting as well as the smallest scale of domestic objects and artefacts that can enhance life during the life span.

Despite the evidence suggesting that space is a key component of any healthcare plan, the design of individual dwellings for encouraging active and healthy aging is a truly underdeveloped area of research, compared, for example, to assistive technologies. Yet, the living space and its impact on quality of life are indeed a very ancient concept [24]. The literature regarding active and healthy aging has in general concluded that healthy life styles through the life span contribute to better quality of life including physical, mental, and cognitive capacity. In general, building and urban design lag behind knowledge from relevant fields such as neuroscience, mental health, rehabilitation, and active healthy aging research. Our aim is to help bridge this gap by creating a learning platform for the relevant disciplines that is experiential and theory driven, while at the same time targeted to generating marketable solutions of the built environment. One area where this leaning platform may be considered of urgent importance is the emerging field of adoption of healthier life style patterns and the need for environmental factors to support these changes.

### 2.2. Life Style Patterns and Environmental Factors to Support Active Healthy Aging (AHA)

“More of the Same Is Not Enough” [25] reviews the most recent research efforts to increase physical activity and thus decrease sedentary life styles, especially among older adults, thus contributing to the top ten chronic diseases worldwide [26]. Understanding how to do more to change this “pandemic” pair of sedentary lifestyles and chronic diseases burden requires new approaches to our theoretical understandings of the causes, as well as the design of innovative interventions to change them at the individual level, since the “one-model-fits-all” approach does not take into account the ecological perspective at the root of the problem. The ecological model for changing toward healthier life styles take into account multiple predisposing factors and barriers such as intrapersonal variables (e.g., personality, health beliefs, knowledge, attitudes, and skills), interpersonal processes, and their likely interactions with genetics as well as community and macro/public policy levels factors [27]. Furthermore, research has shown that interventions for change are likely to take place, not in the conventional healthcare system, but rather in what sociologists have labelled “enabling spaces.” Virtual or physical enabling spaces provide the opportunity for peer learning and teaching that could be actualized within a community. Copresence and observation could provide the mechanism that would generate this learning [28, 29].

The diversity of noncommunicable chronic diseases (NCDs), which hamper healthy and active aging, also shares key modifiable life style factors: sedentary lifestyles with lack of physical activity, poor nutritional habits, high stress levels, and lack of social connectivity [30]. Each one of these modifiable lifestyle factors can be improved with appropriate attention to how neighborhoods/workspaces are designed, how architectural design enhances the creation of enabling spaces for mingling and connecting physically, and how green spaces and walkable communities create more active aging environments. The regional authorities, planning agencies, and healthcare systems working in synergies may be able to create an ecosystem that supports active and healthy lifestyles throughout the life span. This includes industries of food production and distribution as well as boutique culinary facilities catering to aging populations and their physical needs.

A crucial component of this ecosystem is the enabling of movement. This is primarily viewed through the concept of universal design. It is the main concept that has provided solutions for the mobility of the general population, yet so far it presents limitations when neurological or mental disorders are concerned [31]. User involvement from the planning stage of these facilities was one of the key elements of fit for purpose. Similarly, when designing for extra care homes accessibility, user involvement at the planning and remodelling stages was a crucial element of success as it was the involvement of all professionals involved including architects and builders that were aware of universal design and assistive technologies [32]. The next domain that our theoretical model for a multidisciplinary teaching program focuses on is the role of what is now a serious priority for the EIP AHA and WHO and generally falls under the title of age-friendly environment [33, 34].

## 3. Age-Friendly Liveable Environment

### 3.1. Living Styles Supported by Built Environment

The built environment is commonly used in connection with technology supported aging [35]. However, here we would suggest that the WHO definition of environments is more useful in considering healthy and active aging. This definition is broad: “*all the factors in the extrinsic world that form the context of an individual's life; these include home, communities and the broader society; within these environments are a range of factors, including the built environment, people and their relationships, attitudes and values, health and social policies, systems and services*” [1, p. 240]. As it is important for older people to stay at their homes as long as possible [36] some home adaptations might prolong aging in place, the preferred alternative by persons and also a more cost-effective way from a societal perspective [37]. Yet, even if for supported accommodation, research involves physical environment as a determinant of active aging [38] and links QoL with homelike design traits [39, 40] and the fine balance between requirements for safety and homelike environments [41].

In addition to the homelike age-friendly environment, there is an emerging literature of the important connection to nature and its restorative and therapeutic value, along with space for physical and recreative activities in mental health and healthy aging. We discuss these topics next.

### 3.2. Mental Wellbeing and Nature

Research has long established the beneficial elements of nature to mental and physical wellbeing [42, 43], and there is a strong link between perceived sense of health and availability of green areas especially for the elderly and even more so for those living in urban environments [44]. Its integration in the architectural environment of home can have multiple benefits for the resident, from a window view, to the beneficial feel of a breeze and sunshine, which can be appreciated even by patients with Alzheimer's disease [23], to mild form of exercise or gardening, especially if combined with raised accessible flower beds. Yet, gardening does not cover all potential benefits of incorporating nature into design. For instance, in more complex healthcare environments, views to gardens can act as orientation elements [14].

### 3.3. Physical Activity Space

As far as physical activity space is concerned, ergonomic design of healthcare facilities, mainly concentrating on nurses' movement, ignored the complications of confined space for patients without access to the outdoors. Single-loaded corridors for instance could increase the opportunity for walks indoors as well as allow better orientation [21]. At a residential environment, however, there could be other solutions for physical exercise that could be creatively codesigned with appropriately trained architect and the input of the carer and the resident.

### 3.4. Creativity and Social Interaction Spaces

In healthcare environments creativity could be enhanced through a variety of spaces designed for different uses, such as dancing or exercise and space for horticulture, to give to examples out of the numerous possibilities, rather than the one-type-of-common-room-fits-all approach. Research on long-term care connected architecture and the implementation of therapeutic regime through the availability/lack of such areas [45].

### 3.5. Adjusting an Older Residential Care Facility to Contemporary Dementia Care Visions

In residential settings suitable comfortable sitting, presenting a variety of types in accordance with individual preferences and functional elements such as task lighting, worktops, and variety of storage could provide opportunity that a variation of the “student bedsit” that is often applied in care environments, that is, one desk, one bed with a side table, and a wardrobe, or hopefully if it is like a 4- or a 5-star hotel room an additional armchair and a coffee table, could not possibly cover.

### 3.6. Visiospatial Orientation

External views, art, and positive or negative distraction methods [23], such as concealing the external door or using clearly visible clues and colour for bathroom doors, might prove invaluable tools for the orientation and the cognitive function of older people and especially those suffering from dementia.

### 3.7. Autonomy and Independence

**Figure 2:**
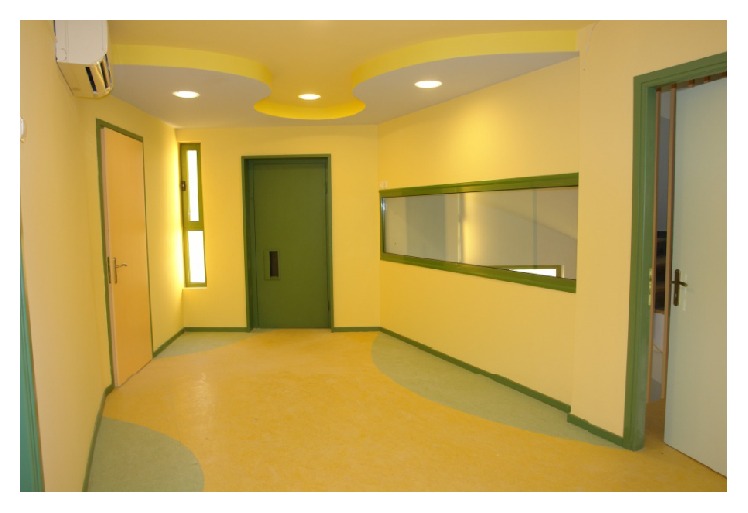
Day Centre for children and adolescents with autism in Paleo Faliro, Greece. Designed by SynThesis Architects.

**Figure 3:**
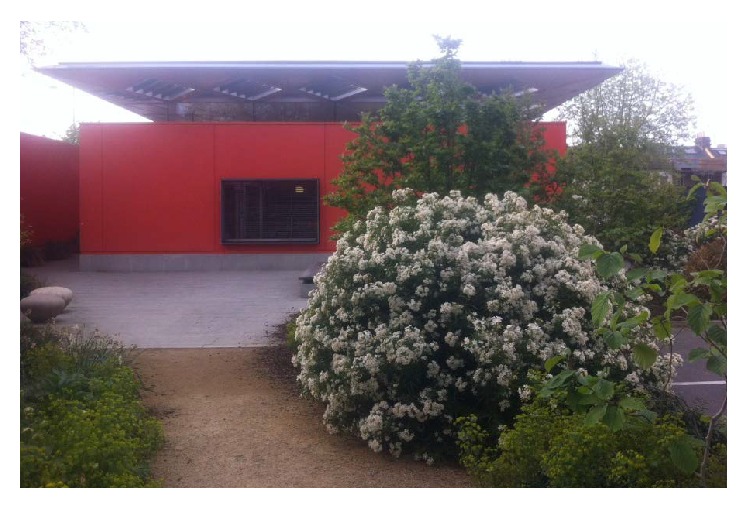
Maggie's West London, located at Charing Cross Hospital. Designed by Rogers Stirk Harbour + Partners.

Control is a factor that tends to appear lower in the pyramid of needs, when compared to more basic needs of sustaining life. However, it is linked with improved health [6], mental healthcare [21], and noninstitutional design frameworks that cater for heterogeneity [40, 46]. At a strategic level, control might be expressed through user involvement and through accessibility means that might enter the core of day-to-day life, such as the case of accessible kitchens or fixtures and fittings at a suitable height. In general, the healthcare environments granted significant control to healthcare professionals as opposed to patients [22]. Yet, lately through increasing patient involvement in the design process we have seen projects that grant significant spatial control to the patients (Figure 2). A very interesting example from the area of cancer care provision is the Maggie's Centre initiative (Figure 3), an innovative, holistic type of facility for cancer patients that has been developed from Maggie Jenks, an architect, when she had been diagnosed with terminal cancer [47].

### 3.8. Fall Prevention Architectural Elements

Lighting, carefully designed circulation spaces, especially in turning points, comfortable and strong grab-rails, preferably cleverly integrated in the decoration rather than the sad and possibly dangerous accessibility devices, opportunities for mind and body exercise for the brain through salutogenic design, and solid and strong furniture at an adequate height for people lifting themselves comfortably are only some of the possibilities that architects can consider as their tools in their aim to design an environment that allows older people to use their body in a safer manner. Yet, the lack of research and the lack of a communication channel between the disciplines have not provided the data that would allow these solutions to be approached systematically and eventually enter the design guidelines documentation allowing their broader implementation.

## 4. Results and Discussion

Based on the theoretical models and research utilizing these models as cited above, we suggest that the creation of an experiential curriculum for all relevant players in designing and building facilities to meet the needs of an aging society is a goal we should be aspiring to. The planning of this type of curriculum should involve all sectors of the economy, that is, governmental, private, and the third sector, as well as cross-industry from the very beginning. This would utilize the experience of the stakeholders, from policy to user level, and enable the burning issues that are well known to those that deal with the subject on an everyday basis to be addressed in a more systematic way. We are aware, for example, of participatory design initiatives such as the Collectively Commissioned Housing in Casteren, Netherlands, where the architects worked mainly as facilitators and residents were the main decision-makers, which proved a cost-effective solution [48]. Such an approach is important for highlighting the importance of the involvement of the users and the financial gains of similar approaches. Yet for more elaborate dwellings, such as residences for vulnerable populations, this crucial amount of user involvement should be complemented by substantial know-how from the architects on special design aspects and implications. For instance, an excellent case of a synergy between informed architectural intervention and the active involvement of users and carers at all levels of decision-making has been the Foyer Élan Retrouvé in Paris, an accommodation facility for the social reintegration of adult mentally ill patients that generated innovation and at the same time presented high user satisfaction results [21, 49]. Similarly, the close collaboration between staff working on a day centre for children with developmental disabilities and a team including a medical architect and a neuroscientist in Faliro, Athens, presented significant reduction of the autistic behaviour of the children during the time they used the facility. In that case, staff would enable a design that was fully compatible with the care regime and the experts introduced to the design elements such as the evidence-based use of positive and negative distraction through colours, shapes, and patterns [50, 51]. Central to these initiatives has been the task to work on an interdisciplinary language and understanding in order to deliver a more evidence based, inclusive practice.

Medical sciences have a clear knowledge that the perception and the physiology of an older frail person differs from a normative one. Yet, that message has not entered the conscious design process of the built environment professionals, as the perception and visual distraction in architectural literature [52] concentrates on architectural-focused instead of person-focused aims, unrelated to the distortions of perception due to ill health. Moreover, research suggests that architects might have a combination of lack of knowledge and misconceptions on the actual needs and preferences of the elderly and research by design projects, such as the example of a Dementia ward at Flanders [45, 53]. This gap of architectural knowledge and education on the perception and users' needs should make us reflect on the way architectural education is delivered as well as potential for further research.

It would be important, among others, for practitioners, designers, and stakeholders, to understand to what extent the built environment is adequate for its residents. It would be important, for instance, to research the safety of universal design features in an older person's bathroom and ways for improvement. Through learning we could encourage designers to broaden their perspective of what constitutes innovative architecture, in a way more human-focused than the glass and steel iconic landmarks. It would be important to help them understand user needs across the lifespan when designing public outdoor areas. That would be the way to create a series of public space improvements that might encourage people to be confident enough and go out of their home in a not so cold day and engage in social activities. A module should provide students the know-how of facility planning in order to create facilities that could act as a magnet for older and frail people and how these could be intergraded in the urban grid. Through case study learning and fieldtrips they could familiarise themselves with existing examples of good practice that need to be explored and lessons to be learned. They should be able to understand the limitations of the existing definition of accessibility in covering the needs of the largest ever generation approaching old age. It would be also important for all stakeholders to build the skills to comprehend the value of more elaborate and neuroscientific and technologically sophisticated interventions that could generate significant health benefits with relatively low costs. The list is indicative and the authors could by no means cover the whole spectrum of research possibilities and the learning potential in a single paragraph.

### 4.1. Conclusions

Part of the gap between human science and architecture relates to the educational process of architects, the lack of evidence-based guidelines or best practices that architects could refer to, and the lack of translational research across fields. The article presented a theoretical framework for incorporating into architecture the vision of design for active and healthy living; features medical research suggest would add value toward active and healthy aging.

The challenges aging presents to individuals and society as a whole are complex and multilevel. For example, aging person functionality is impacted by not only one's intrinsic personality but also by ecopsychosocial elements. These multilevel and complex societal domains can be modified through the creation of multidisciplinary knowledge and educational opportunities along with design for wellbeing research to explore the benefits of interlinking space, architecture of living environments, and their impact on physical and cognitive functionality. It is important for medical practitioners to be aware of the developments and opportunities in the built environment and vice versa and at the same time to see the social impact and the extent in terms of social scale of the problem. Creating a multidisciplinary program for the various professionals (architects, doctors, and planners) and aging persons can be accomplished through blended learning curriculum (see, e.g., http://www.eitdigital.eu/eit-digital-academy/master-school/). The EIP AHA through its synergies initiative can play an important leadership role for securing funding to initiate such blended learning environments throughout the EU.
